# lncRNA NTT/PBOV1 Axis Promotes Monocyte Differentiation and Is Elevated in Rheumatoid Arthritis

**DOI:** 10.3390/ijms19092806

**Published:** 2018-09-18

**Authors:** Chin-An Yang, Ju-Pi Li, Ju-Chen Yen, I-Lu Lai, Yu-Chen Ho, Yu-Chia Chen, Joung-Liang Lan, Jan-Gowth Chang

**Affiliations:** 1 Department of Laboratory Medicine, China Medical University Hospital, Taichung 40447, Taiwan; yangginan81@gmail.com; 2 Division of General Pediatrics, China Medical University Children’s Hospital, Taichung 40402, Taiwan; 3 College of Medicine, China Medical University, Taichung 40402, Taiwan; d888203@gmail.com (J.-P.L.); tw.lesswind@gmail.com (Y.-C.H.); jounglan@me.com (J.-L.L.); 4 Center for Precision Medicine, China Medical University Hospital, Taichung 40447, Taiwan; t92989@mail.cmuh.org.tw; 5 Rheumatism Research Center, China Medical University Hospital, Taichung 40447, Taiwan; 6 Division of Rheumatology and Immunology, China Medical University Hospital, Taichung 40447, Taiwan; 7 Epigenome Research Center, China Medical University Hospital, Taichung 40447, Taiwan; t24399@mail.cmuh.org.tw (J.-C.Y.); vero0214@gmail.com (I.-L.L.)

**Keywords:** long noncoding RNA, NTT, C/EBPβ, PBOV1, monocyte differentiation, rheumatoid arthritis

## Abstract

Monocytes/macrophages are important in orchestrating inflammatory responses. However, knowledge of the long noncoding RNA (lncRNA) regulation of monocytic cell differentiation and diseases remains limited. We aimed to elucidate the role of the 17 kb lncRNA noncoding transcript in T cells (*NTT*) in monocyte functions. Knockdown and chromatin immunoprecipitation (ChIP) assays in THP-1 cells (human monocytic leukemia cell line) revealed that *NTT* is regulated by the monocyte key transcription factor C/EBPβ and that it binds to the promoter of nearby gene *PBOV1* via hnRNP-U. Overexpression of *PBOV1* in THP-1 cells resulted in cell cycle G1 arrest, differentiation into macrophages, a marked increase in *IL-10* and *CXCL10* mRNA levels, and upregulation of the costimulatory molecules. In contrast to the downregulated *NTT* observed in lipopolysaccharide (LPS)-treated THP-1 cells, the *C/EBPβ/NTT/PBOV1* axis was found to be hyperactivated in peripheral blood mononuclear cells (PBMCs) of first-time diagnosed untreated early rheumatoid arthritis (RA) patients, and their gene expression levels decreased markedly after treatment. Higher initial *C/EBPβ/NTT/PBOV1* expression levels were associated with a trend of higher disease activity DAS28 scores. In conclusion, our study suggests that the lncRNA *NTT* is a regulator of inflammation in monocytes, and its activation participates in monocyte/macrophage differentiation and the pathogenesis of RA.

## 1. Introduction

Monocytes are central players in orchestrating complex immune responses. Upon sensing stimuli from infectious or aseptic sources, monocytes can differentiate into dendritic cells (DCs) or macrophages [1]. Being trafficked to the sites of inflammation, macrophages exert their functions by phagocytosis, cytokine secretions, and interactions with cells of adaptive immunity, and their functions may change along the progression of inflammation [2,3]. Macrophage differentiation and polarization to pro-inflammatory or immunosuppressive phenotypes are tightly regulated by environmental factors (i.e., cytokines, metabolic stresses), transcription factors, and noncoding RNAs [2,4]. Among noncoding RNAs, micro-RNAs (miRNAs) have been extensively studied as mediators that intricately regulate multiple functions of monocytes/macrophages [5,6]. Dysregulations of miRNAs has been implicated in a variety of diseases, including infection, atherosclerosis, obesity, cancer, and autoimmune/autoinflammtory diseases [5,7]. Long noncoding RNAs (lncRNAs) are noncoding RNAs that are more than 200 nucleotides long. Accumulated evidence has shown that lncRNA can affect different stages of gene regulation via RNA–DNA, RNA–RNA, or RNA–protein interactions [8]. Similarly, lncRNAs have been reported to fine-tune both innate and adaptive immune responses [9,10,11,12,13]. However, knowledge of the mechanisms of lncRNA regulation for monocyte differentiation/activation and their roles in inflammatory diseases remains limited.

It was presented by Satpathy A.T. and Chang H.Y. in Immunity 2015 that several lncRNAs are involved in the differentiation of myeloid cells and their activations after stimulation with different Toll-like receptor (TLR) ligands [14]. *Lnc-DC* was reported to be upregulated by the transcription factor PU.1 during monocyte differentiation to classical dendritic cells (cDCs), while *HOTAIRM1* was found to participate in terminal granulocyte differentiation [15,16]. Upon TLR2 stimulation, *lincRNA-Cox2* has been demonstrated to regulate immune gene transcriptions via interactions with the heterogeneous nuclear ribonucleoproteins (hnRNP)-A/B and hnRNP-A2/B1, while *THRIL* was shown to regulate TNF-α production via interaction with hnRNP-L in THP-1 macrophages [17,18]. Noncoding transcript in T cells (*NTT*) is a 17 kb lncRNA which is expressed in the nucleus. It was discovered in 1997 in activated CD4+ T cells and in peripheral blood mononuclear cells (PBMCs) stimulated with HIV peptides, suggesting its role in adaptive immunity [19,20]. Yet, the mechanisms underlying the *NTT*-mediated potential regulation of T-cell functions are still unclear. Furthermore, whether and how *NTT* is involved in innate immunity are currently unknown. 

*NTT* is located at chromosome 6q23–q24, which is close to several genes related to immune function, hematopoiesis, and cell proliferation, including *IFNGR1, TNFAIP3, MYB*, and *PBOV1* [20]. *IFNGR1* is associated with macrophage TNF-α production and susceptibility to *Listeria monocytogenes* infection [21]. *TNFAIP3* is an essential negative regulator of inflammation, and dysregulation of *TNFAIP3* is associated with autoimmune diseases [22]. *MYB* is a transcription factor reported to regulate myeloid hematopoiesis and usually is aberrantly expressed in leukemia [23]. Similarly, *PBOV1* has been reported to be overexpressed in breast, prostate, and ovarian cancer tissues, regulating tumor proliferation; however, the function of *PBOV1* on immune cells has not been discovered [24,25,26,27]. Due to its large size (17 kb nt) and proximity to these potentially immune-related genes, it has been suggested that *NTT* might exert its function via regulating the nearby genes [20]. 

In this study, we aimed to investigate the role of the lncRNA *NTT* in monocyte functions, how *NTT* is regulated in inflammation, and its potential dysregulation in a chronic inflammatory autoimmune disease, rheumatoid arthritis (RA). 

## 2. Results

### 2.1. NTT (Noncoding Transcript in T Cells) Is Expressed in Human Monocytic Cells and Is Regulated by C/EBPβ

**Figure 1:**
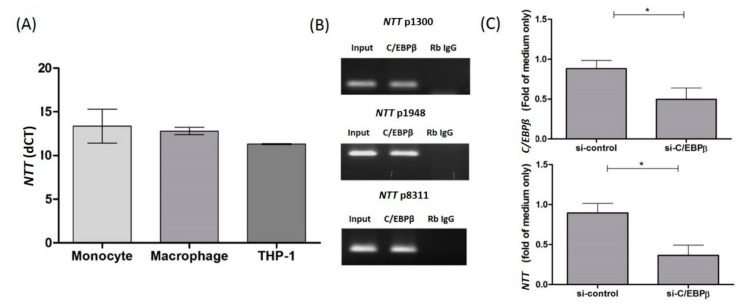
Expression of noncoding transcript in T cells (*NTT*) in immune cells and regulation of *NTT* during lipopolysaccharide (LPS) stimulation. (**A**) *NTT* is expressed in human primary monocytes, monocyte-derived macrophages, and THP-1 cells; *n* = 3, bars represent mean ± SEM. (**B**) THP-1 cell nuclear lysates were fixed and immunoprecipitated by anti-C/EBPβ or isotype control IgG antibody (ChIP), and the antibody-bound DNA sequences of three positions of *NTT* promoter were detected by PCR and gel electrophoresis; here is a representative graph of three independent experiments, showing binding of C/EBPβ. (**C**) Knockdown of C/EBPβ in THP-1 cells by si-RNA resulted in lower expression levels of *NTT*; *n* = 5, bars represent mean ± SEM. *: *p* < 0.05 by Wilcoxon signed rank test.

*NTT* was found to be expressed in resting human primary monocytes, monocyte-derived macrophages, and the THP-1 cell line (Figure 1A). By examining the promoter sequence of *NTT*, we identified the potential binding motif of C/EBPβ, a key transcription factor in monocytes. To further check if C/EBPβ binds to the *NTT* promoter and regulates *NTT* expression, we performed a chromatin immunoprecipitation assay (ChIP) and siRNA knockdown for C/EBPβ in the THP-1 cell line. C/EBPβ binding was detected on three positions of the *NTT* promoter (Figure 1B), and C/EBPβ knockdown in THP-1 resulted in decreased *NTT* expression (Figure 1C). 

### 2.2. NTT Regulates Downstream Gene PBOV-1 via HnRNP-U Binding

**Figure 2:**
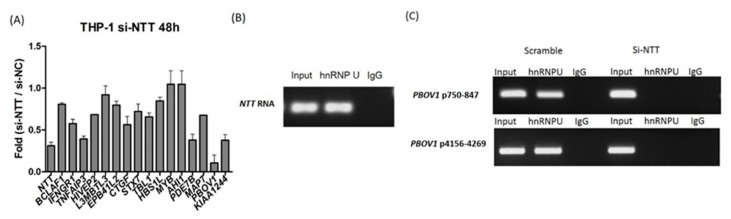
*NTT* acts in *cis* on genes at the local proximity. (**A**) Knockdown of *NTT* in THP-1 cells resulted in decreased expression levels of downstream genes, with the most prominent effect on *PBOV1*; *n* = 3, bars represent mean ± SEM. (**B**,**C**) *NTT* regulates *PBOV1* via binding with hnRNP-U to the *PBOV1* promoter. (**B**) *NTT* RNA binds to hnRNP-U as detected by RNA IP; representative graph from three independent experiments. (**C**) DNA ChIP assay detecting the promoter sequence of *PBOV1* (2 positions) using anti-hnRNP U antibody in THP-1 cells transfected with si-RNA for *NTT* or scramble control; representative graph from three independent experiments.

Next, we investigated how the large lncRNA *NTT* regulates nearby genes (potentially downstream genes). *NTT* expression in THP-1 was knocked down by si-RNA and the relative mRNA levels of nearby genes were analyzed (Figure 2A). Transfection of si-*NTT* in THP-1 led to decreased expression in almost all downstream genes, except *L3MBTL3*, *MYB*, and *AHI1. NTT* knockdown showed the greatest impact on *PBOV1* expression (Figure 2A). Since it has been reported that lncRNAs may bind to hnRNP proteins to regulate downstream genes, we further studied if *NTT* could bind to the *PBOV1* promoter via interaction with the hnRNP-U protein. An RNA immunoprecipitation assay showed that *NTT* could bind to hnRNP-U (Figure 2B). DNA ChIP showed that hnRNP-U binds to two positions of the *PBOV1* promoter, and the hnRNP-U binding became undetectable after *NTT* knockdown (Figure 2C), suggesting *NTT* might enhance *PBOV1* expression by interacting with hnRNP-U binding to the promoter of *PBOV1*.

### 2.3. C/EBPβ, NTT, and PBOV1 Expression Levels Were Highly Elevated in Fresh Rheumatoid Arthritis (RA) Patients

**Figure 3:**
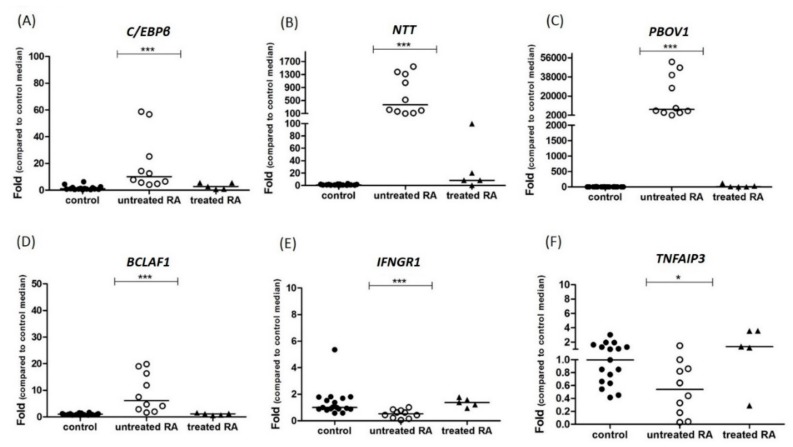
*C/EBPβ/NTT/PBOV1* expression levels are elevated in peripheral blood mononuclear cells (PBMCs) of untreated rheumatoid arthritis (RA) patients. RNA expression levels of (**A**) *C/EBPβ*, (**B**) *NTT*, (**C**) *PBOV1*, (**D**) *BCLAF1*, (**E**) *IFNGR1* and (**F**) *TNFAIP3* in PBMCs derived from healthy controls (*n* = 17), untreated RA patients (*n* = 10), and treated RA patients (*n* = 5, blood drawn after 2 years of follow-up). Lines represent medians; * *p* < 0.05, *** *p* < 0.0001 by Kruskal–Wallis tests. RNA expression levels are shown as folds compared with median levels of healthy controls.

In order to understand the potential roles of *NTT* in inflammatory diseases, we evaluated the expression levels of *C/EBPβ*, *NTT*, and downstream genes in PBMCs derived from first-time diagnosed, untreated RA patients. We were able to follow up with 8 of the 10 patients, and blood was drawn again in 5 of the 8 patients after 2 years of RA treatment. As compared with the levels detected in healthy control PBMCs, *C/EBPβ*, *NTT*, and *PBOV1* expressions were significantly elevated in freshly diagnosed RA patients (median folds for *C/EBPβ* = 10.13, *NTT* = 369.76, *PBOV1* = 7455.33), and the levels markedly decreased after 2-year treatment (Figure 3A–C). The expression of another *NTT* downstream gene, *BCLAF1*, showed the same trend as *PBOV1* but with a much lower fold (Figure 3D). In contrast, as for other *NTT*–downstream immune-related genes, *IFNGR1* and *TNFAIP3*, the expression levels decreased in freshly diagnosed RA patients (Figure 3E–F). 

### 2.4. Association of C/EBPβ, NTT, and PBOV1 Expression Levels with RA Disease Severity

**Figure 4:**
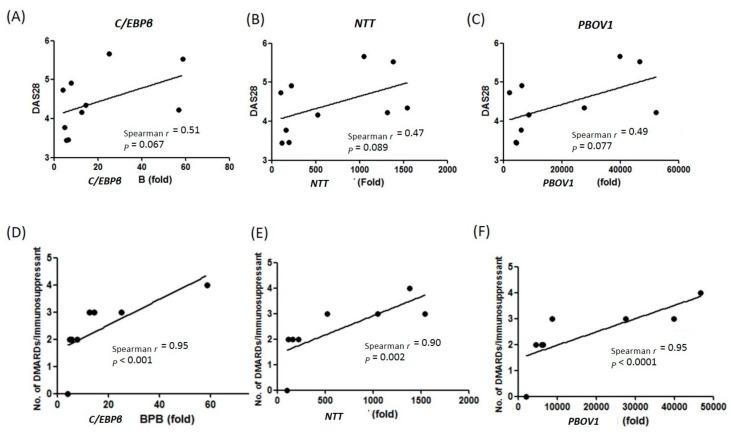
Correlations of *C/EBPβ/NTT/PBOV1* expression levels with RA disease severity score DAS28 (**A**–**C**) and usage of multiple disease-modifying antirheumatic drugs (DMARDs)/immunosuppressant (**D**–**F**). RNA expression levels are shown as folds compared with median levels of healthy controls.

As shown in Table 1, *C/EBPβ/NTT/PBOV1* expression levels did not associate with levels of initial laboratory inflammatory markers, including rheumatoid factor (RF), anticyclic citrullinated peptide antibody (anti-CCP), erythrocyte sedimentation rate (ESR), and C-reactive protein (CRP). In addition, *C/EBPβ/NTT/PBOV1* expression did not correlate with hemoglobin (Hb), IL-1β, TNFα, and IL-6 levels (Table S1). However, we found a trend of positive correlation between *C/EBPβ/NTT/PBOV1* expressions and initial disease activity scores (DAS28) (Table 1, Figure 4A–C). Linear regression analysis showed that *C/EBPβ* expression level positively correlated with the simplified disease activity index (SDAI) (*r*^2^ = 0.54, *p* = 0.015, Table 1). Furthermore, higher *C/EBPβ/NTT/PBOV1* levels positively correlated with the usage of multiple disease-modifying antirheumatic drugs (DMARDs)/immunosuppressants at the 2-year follow-up (Table 1, Figure 4D–F). Only one RA patient (in the initial *C/EBPβ/NTT/PBOV1*-high group, patient No. 3) required a biological agent (Adalimumab) for treatment. All patients except the one with the highest initial *C/EBPβ/NTT/PBOV1* levels (Table 1, patient No. 1) had lower than 2.7 DAS28 scores after 2 years of RA treatment. Collectively, these results suggest that the *C/EBPβ/NTT/PBOV1* axis is highly activated in untreated RA patients, and higher expression levels might be associated with a higher disease inflammatory status, which requires more medications to control. 

### 2.5. Overexpression of PBOV1 in THP-1 Cells Led to Cell Cycle Arrest and Differentiation to Macrophages

**Figure 5:**
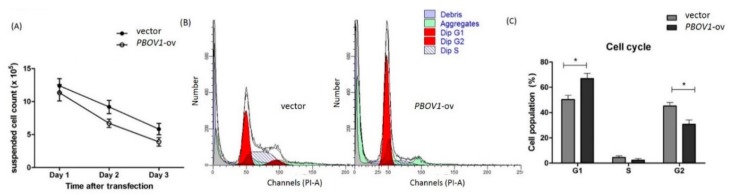
Overexpression of *PBOV1* in THP-1 cells results in cell cycle arrest. (**A**) Count of suspended THP-1 cells after transfection of *PBOV1*-overexpression vectors; *n* = 6, bars represent mean ± SEM. (**B**) Cell cycle analysis of THP-1 cells transfected with control vector (left) or with *PBOV1*-overexpression vector (right) for 48 hours; here are representative graphs of four independent experiments, showing G1 arrest in *PBOV1*-overexpressing THP-1. (**C**) Percentage of THP-1 cells in Gap 0/Gap 1 (G0/G1), Synthesis (S), and Gap 2/Mitosis (G2/M) phase; *n* = 4, bars represent mean ± SEM. * *p* < 0.05 by Mann Whitney U test.

**Figure 6:**
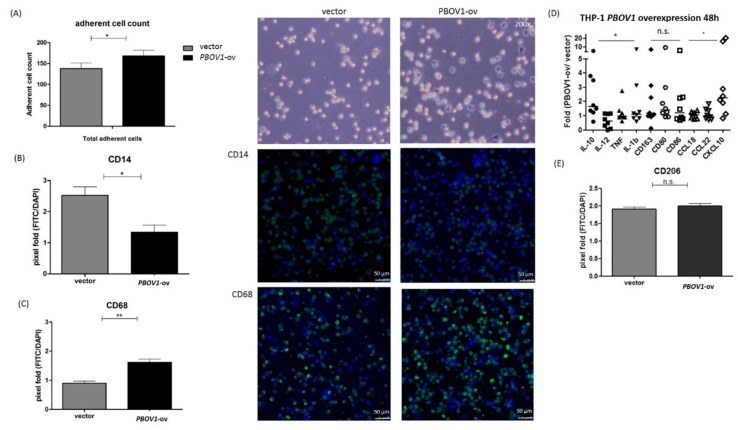
Overexpression of *PBOV1* in THP-1 cells promotes differentiation into macrophages. (**A**) Adherent cell counts increased after transfection of *PBOV1* overexpression vector for 48 h. Left: Bars represent mean ± SEM, *n* = 6; Right: images under light microscope after removing suspended cells; * *p* < 0.05 by Mann–Whiney U test. (**B**) Decreased CD14 expression in *PBOV1*-overexpressing THP-1 cells. Left: Columns are CD14-FITC-positive cells counted by Image J tool, bars represent mean ± SEM, *n* = 5; Right: confocal microscopy images of immunohistochemical staining of CD14 (green) and cell nucleus (blue). * *p <* 0.05 by Mann Whitney U test (**C**) Increased CD68 expression in *PBOV1*-overexpressing THP-1 cells. Left: Columns are CD68-FITC-positive cells counted by Image J tool, bars represent mean ± SEM, *n* = 5; Right: confocal microscopy images of immunohistochemical staining of CD68 (green) and cell nucleus (blue). ** *p <* 0.01 by Mann Whitney U test (**D**) M1/M2 profiling of THP-1 cells transfected with *PBOV1*-overexpression vector for 48 h. Lines represent medians, *n* = 8; showing marked elevation in IL-10 and CXCL10 RNA expressions; * *p* < 0.05 by Kruskal–Wallis test; *n.s*.: not significant by Kruskal–Wallis test (**E**) *PBOV1*-overexpressed THP-1 cells secrete more CXCL10 in the supernatant than control. Bars represent mean ± SEM, *n* = 6; * *p* < 0.05 by Mann–Whiney U test. (**F**) THP-1 cells transfected with control vector or *PBOV1*-overexpression vector were further differentiated into macrophage (via phorbol 12-myristate 13-acetate (PMA)) and polarized to M2 (via IL-4 and IL-13). Immunohistochemical staining of CD206 shows no difference of expression between THP-1 transfected with control vector or with *PBOV1*-overexpression vector; *n* = 5, bars represent mean ± SEM. *n.s.*: not significant by Mann Whitney U test.

Although marked *PBOV1* upregulation was observed in the PBMCs of freshly diagnosed RA patients, the function of *PBOV1* in monocytes remains unknown. Therefore, we transfected the *PBOV1*-overexpression vector to the THP-1 monocytic cell line for 48 h. As compared with cells transfected with the control vector, *PBOV1*-overexpressing THP-1 cells showed a lower suspended cell count (Figure 5A) but a more adherent cell count (Figure 6A). Furthermore, a higher proportion of cells arrested at the G1 phase of the cell cycle was found in *PBOV1*-overexpressing THP-1 cells (Figure 5B–C). Further immunohistochemical staining of the adherent cells showed lower monocyte marker CD14 and higher macrophage marker CD68 expressions in *PBOV1*-overexpressed THP-1 cells as compared with controls, suggesting differentiation toward macrophages (Figure 6B–C).

**Figure 7:**
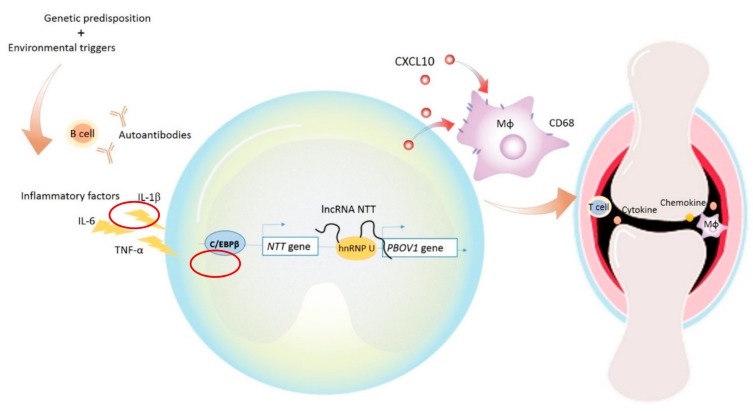
Graphical summary showing the proposed model of the roles of *C/EBPβ/NTT/PBOV1* upregulation in early RA pathogenesis. RA pathology could be induced by environmental triggers in genetically predisposed individuals. Autoantibodies produced by B cells and inflammatory cytokines such as IL-6, IL-1β, and TNF-α appear in the disease process. Perhaps under the stimulation of these factors, the transcription factor C/EBPβ binds to the promoter of *NTT*, leading to the transcription of *NTT*. *NTT* binds to the promoter of *PBOV1* via hnRNP-U, then the enhanced expression of *PBOV1* promotes monocyte/macrophage differentiation and the secretion of cytokines and chemokines including CXCL10. CXCL10 can promote the migration of immune cells to the site of inflammation, such as the synovial joints in RA.

In order to investigate whether *PBOV1* also affects macrophage polarization, mRNAs of M1/M2 related cytokines, cell surface markers, and chemokines were analyzed in *PBOV1*-overexpressed THP-1 cells (Figure 6D). The anti-inflammatory cytokine IL-10 mRNA level was found to be elevated in *PBOV1*-overexpressed THP-1 cells, while the levels of pro-inflammatory cytokines IL-12, TNF-α, and IL-1β were not (Figure 6D). Conversely, the pro-inflammatory chemokine CXCL10 mRNA level was found to be markedly upregulated in *PBOV1*-overexpressed THP-1 cells, while the levels of the anti-inflammatory chemokines CCL18 and CCL22 remained unchanged (Figure 6D). The mRNA levels of both M1 (CD80/CD86) and M2 (CD163) related cell markers were increased in cells overexpressing *PBOV1* (Figure 6D). We then evaluated the IL-10 and CXCL10 protein levels in supernatants of THP-1 cells transfected with the *PBOV1*-overexpression vector or control vector for 48 h. However, only elevated secretion of CXCL10 was observed in *PBOV1*-overexpressed THP-1 cells (Figure 6E). IL-10 protein was undetectable in all supernatants (data not shown). Further polarization of THP-1 cells to M2 macrophages using sequential stimulations with phorbol 12-myristate 13-acetate (PMA), IL-4, and IL-13 revealed no difference of CD206 protein expression between *PBOV1*-overexpressing cells and cells transfected with the control vector (Figure 6F). Taken together, *PBOV1* overexpression in THP-1 cells promoted differentiation to macrophages and increased CXCL10 secretion. CXCL10 could potentially mobilize more immune cells, which then may be trafficked to the site of inflammation together with the differentiated macrophages. The graphical summary showing the proposed model of the roles of *C/EBPβ/NTT/PBOV1* upregulation in RA pathogenesis is shown in Figure 7.

## 3. Discussion

As the central player in orchestrating complex immune responses, monocytes/macrophages are equipped with the ability to react to different stimuli dynamically [2]. Although data are still limited, lncRNAs have been reported to be the link connecting signals transduced by transcription factors and the activation or repression of downstream genes in macrophages [8,14,17,28]. Here, we report that *NTT* is expressed in human monocytes/macrophages and is highly upregulated (around 100- to 1000-fold of normal control) in the PBMCs of untreated RA. While being regulated by the transcription factor C/EBPβ, *NTT* acts in *cis* on gnomically closely located genes related to differential immune responses and macrophage differentiation, adding a layer of complexity to the fine tuning of monocyte functions.

Previous researchers have identified differentially expressed lncRNAs in PBMCs and synovial fibroblasts in patients with RA, a chronic inflammatory joint disease [29,30,31,32]. While the functions of most of these lncRNAs remains unknown, Hotair has been reported to enhance the migration of activated macrophages, thereby participating in RA pathogenesis [31]. In this study, *NTT* was found to be highly elevated in the PBMCs of untreated RA and correlated with the requirement of multiple DMARDs/immunosuppressants during 2 years of treatment. In early RA, *NTT* is possibly upregulated by the transcription factor *C/EBPβ*, which is known to drive myeloid-derived suppressor cell (MDSC) differentiation and the polarization of the more regulatory M2 macrophages [2,33,34,35]. The increased amount of *NTT* bound to the promoter of the downstream gene *PBOV1* via hnRNP-U, and it upregulated *PBOV1* expression to an average of 18,000-fold as compared with healthy controls. These results are consistent with the report that lncRNAs could elevate downstream gene expression via increasing mRNA stability by interacting with hnRNP-U; however, we could not rule out the possibility that *NTT* might also exert its *cis*-action on *PBOV1* via other mechanisms, such as binding to histone modifying complexes [36,37].

Prostate and breast cancer overexpressed 1 (*PBOV1*) was first described to be upregulated in certain cancers involving tumor proliferation and is variably associated with patient survival [24,25,27]. In prostate cancer cell lines, *PBOV1* overexpression was found to promote tumor proliferation and cell cycle progression [25]. However, the role of *PBOV1* in autoimmune diseases is still unknown. We are the first to investigate the function of *PBOV1* in monocytes. As observed in untreated RA PBMCs, high levels of *PBOV1* might be downstream of *C/EBPβ*/*NTT* hyperactivations. Although *C/EBPβ* is known to drive macrophages towards an anti-inflammatory phenotype [33,34], we found that macrophages differentiated from *PBOV1*-overexpressed THP-1 cells upregulated the RNA expressions of IL-10 (mostly between 1- and 2-fold) and the pro-inflammatory chemokine CXCL10 (mostly more than 2-fold). We could not detect IL-10 protein in the supernatant at 48 or 64 h after transfection, suggesting the detection of IL-10 RNA upregulation might be nonspecific and transient. As for CXCL10, markedly elevated protein levels were also detected in *PBOV1*-overexpressed THP-1 supernatants. The pro-inflammatory chemokine CXCL10 has been reported to attract activated macrophages, T cells, and NK cells to the site of inflammation and is upregulated in autoimmune diseases, including RA [38,39,40,41,42]. It is suggested that CXCL10 increases the migration of inflammatory cells via CXCR3-mediated ERK activation and stimulates the production of osteoclastogenic cytokines in CD4+ T cells, resulting in bone destruction in RA [43]. We therefore speculate that *NTT*-induced monocyte/macrophage *PBOV1* expression in genetically predisposed individuals plays a pathogenic role in RA via enhancing the secretion of the chemokine CXCL10, which could lead to synovial inflammation and joint destruction (Figure 7). Studies on the expressions of *NTT* and *PBOV1* in blood monocytes and synovial macrophages of different disease phases might help to clarify their pathogenic roles in RA. Since *NTT* is also expressed in T cells, the function of *NTT* in regulating macrophage—T cell interactions in RA development warrant further research.

## 4. Materials and Methods

### 4.1. Patient Subjects

Peripheral blood mononuclear cells (PBMCs) from freshly diagnosed rheumatoid arthritis patients and sex- and age-matched healthy controls were collected. RNA was extracted by the TRIzol method, further reverse transcribed to cDNA, and tested for the relative expression levels of *NTT* and upstream and downstream genes. This study was approved by China Medical University Hospital Research Ethics Committee on 05 March 2016 (CMUH105-REC2-006, for project “Exploring the regulation and function of a long non-coding RNA *NTT* in inflammation”).

### 4.2. THP-1 Cell Culture, Differentiation, and Polarization Assays

THP-1 cells were grown in LPS-free complete RPMI medium containing 10% fetal bovine serum at 37 °C in an incubator with 5% CO_2_. In M2-polarization experiments, THP-1 cells were differentiated into macrophages using 50 µM PMA for 5 days; THP-1-derived macrophages were then polarized to M2 via treatment of 20 ng/mL IL-4 and 20 ng/mL IL-13 (R&D Systems, Minneapolis, MN, USA) for 2 days.

### 4.3. Isolation of Primary Immune Cells

Human primary T cells and monocytes were isolated from healthy subjects’ peripheral blood by using RosetteSep human T cell enrichment cocktail and RosetteSep human monocyte enrichment cocktail (Stemcell technologies, Vancouver, BC, Canada), followed by Ficoll Paque gradient centrifugation. Macrophages were further derived from monocytes via replacing half of the culture medium each day for 7 days.

### 4.4. Chromatin Immunoprecipitation Assay (ChIP)

THP-1 cells were crosslinked with 0.4% formaldehyde and the reaction was stopped by adding glycine. Fixed cells were lysed according to magnetic chromatin immunoprecipitation kit protocol. The nuclear fractions were sonicated to reduce DNA length to 200–500 bp. The chromatin extract was incubated with magnetic beads and either anti-C/EBPβ/anti-hnRNP-U/anti-ATF3 (all from Abcam, Cambridge, UK) or anti-IgG (negative control) antibody. Another portion of sheared DNA were prepared for making an input control. After immunoprecipitation, the immune complex was eluted, the protein was unlinked, and the associated DNA fragments were purified according to the manufacturer’s instructions (Active Motif, Carlsbad, CA, USA). Finally, PCR of the purified DNA using primers for the respective promoter sequences were performed.

### 4.5. Electroporation for Knockdown and Overexpression Assays 

THP-1 was transfected with 10 nM si-RNAs for *C/EBPβ*, si-RNA for *NTT*, or negative control-siRNA (all purchased from MDBio, Taipei, Taiwan) by using electroporation. Briefly, 5 × 10^6^ cells were resuspended in 500 µL serum-free RPMI medium and incubated with 150 nM siRNA in electroporation cuvettes for 5 min on ice. Cells were then electroporated at 220 mV and 950 µF using Bio-rad Gene Pulser (Midland, ON, Canada) according to manufacturer’s instructions. For *PBOV1*-overexpression assays, THP-1 cells were transfected with 10 µg/mL pCMV6-Entry vector cloned with *PBOV1* or the control vector (both vectors were purchased from OriGene, Rockville, MD, USA) for 48 h. Subsequently, RNAs were extracted for real-time PCR analysis, and culture supernatants were collected for IL-10 (R&D Systems) and CXCL10 (R&D Systems) ELISA analyses.

### 4.6. Reverse-Transcriptase PCR (RT-PCR)

Nuclear and cytoplasmic cell lysates were extracted using the Nuclear/Cytoplasmic Extraction kit (Thermo Fisher Scientific, Waltham, MA, USA) and followed by TRIzol RNA extraction. Two micrograms of RNA were reverse-transcribed using reverse transcriptase and buffers. The resulting cDNA underwent quantitative real-time PCR analysis. The house-keeping gene *GAPDH* was used as the internal control for total and cytoplasmic cell fraction, while *U2* snRNA gene was used as the internal control for nuclear cell fraction.

*NTT* promoter primer sequences:

Position 9501–9746: forward 5′-TGGTATGAACAAGGCAGCAG-3′, reverse 5′-CCTTGTTTGTGCCCATCTCT-3′; position 6846–7059: forward 5′-GGGTGAAAGCAGCCTGTG-3′, reverse 5′-CAGAACAAAAAGAACCCCTGA-3′; position 5825–6095: forward 5′-TCTCCAATCCCCTCTGTGAC-3′, reverse 5′-GAAGGCTATGGGCACTTTCA-3′; position 8204–8425: forward 5′-TCAACATATGCCTTACCTCACA-3′, reverse: 5′-TACCAGGAGCTTGGGATGTT-3′

Gene primer sequences:

*NTT* forward 5′-CTTGGCCTAAAAGGGGATG-3′, reverse 5′-GCACCTTTGGTCTCCTTCAC-3′; *IFNGR1* forward 5′-CATGCAGGGTGTGAGCAG-3′, reverse 5′-AACATTAGTTGGTGTAGGCACTGA-3′; *TNFAIP3* forward 5′-TGCACACTGTGTTTCATCGAG-3′, reverse 5′-ACGCTGTGGGACTGACTTTC-3′; *HIVEP2* forward 5′-CGGCAAGCTTACATCATCAA-3′, reverse: 5′-AGGACGCATCAGGTTTCATC-3′; *PBOV1* forward 5′-GAAAAAGATTCTCATCACTCAAC-3′, reverse 5′-GGTTCTCAAACAGCCTTCC-3′; *C/EBPβ* forward 5′-CGGGCTCAGGAGAAACTTTA-3′, reverse 5′-GGGGTGGCCGCTATTAGT-3′; *GAPDH*: forward 5′-AGCCACATCGCTCAGACAC-3′, reverse 5′-GCCCAATACGACCAAATCC-3′; *U2* snRNA: forward 5′-TTTGGCTAAGATCAAGTGTAGTATCTGTTC-3′, reverse 5′-AATCCATTTAATATATTGTCCTCGATAGA-3′

### 4.7. Cell Cycle Analysis

Transfected THP-1 cells were collected and fixed in 70% ethanol at −20 °C overnight. Fixed cells were washed and labelled with propidium iodide (Sigma-Aldrich, St. Louis, MO, USA) in the presence of RNase A (Sigma-Aldrich) and Triton X-100 for 30 min in the dark. FACSCanto flow cytometer and the ModFit LT program were used to analyze the percentages of cells within each phase of the cell cycle.

### 4.8. Immunofluorescence Staining

Monolayer of THP-1 cells was attached to slides using the Cytospin centrifugation method. Cells were fixed by 100% methanol and stained with primary antibodies (anti-CD14, anti-CD68, anti-CD206, all from Abcam, Cambridge, UK) at 4 °C overnight after blocking with PBS (phosphate buffered saline) containing 1% (wt/vol) fetal bovine serum (FBS). Cells were then washed and stained with fluorochrome-conjugated secondary antibodies. DAPI (4′,6-diamidino-2-phenylindole) nuclear stain was applied. Slides were further analyzed under a confocal laser scanning microscope. Image J software was used to quantify the relative amount of positive staining.

### 4.9. Statistical Analyses

Mann–Whitney U test was applied to compare the percentage of macrophage marker expression between THP-1 cells with or without transfection of *PBOV1*-overexpression vector. Kruskal–Wallis test was used to compare the expression levels of *NTT* and related genes in healthy subjects, fresh RA patients, and treated RA patients. Spearman’s test was performed to analyze the correlation between *NTT* expression level and RA disease activity. All statistical tests were performed on GraphPad Prism version 5 (La Jolla, CA, USA).

## 5. Conclusions

This study suggests that the lncRNA *NTT* is a regulator of inflammation in monocytes, and its activation participates in monocyte/macrophage differentiation via *PBOV1* upregulation. Hyperactivation of the C/EBPβ/*NTT*/*PBOV1* axis might play a role in the pathogenesis of RA via enhanced secretion of the proinflammatory chemokine CXCL10.

## Figures and Tables

**Table 1:** Clinical characteristics of RA (rheumatoid arthritis) patients and their *C/EBPβ/NTT/PBOV1* expression levels in PBMCs (peripheral blood mononuclear cells).

Basic Information			Initial Lab Data				Initial Disease Activity		RNA Expression at RA Diagnosis			Clinical Parameters at 2-Year Follow-Up	
Patient No.	Sex	Age	RF (IU/mL)	Anti-CCP (U/mL)	ESR (mm/h)	CRP (mg/dL)	DAS28	SDAI	C/EBPβ (fold)	NTT (fold)	PBOV1 (fold)	Number of Medications Used in Addition to NSAID	DAS28
1	F	57	28.2	29	44	1.15	5.53	19.7	58.69	1379.57	46,663.28	4 (Hydroxychloroquine, Sulfasalazine, Cyclosporine, Prednisolone)	3.86
2	F	56	688	2.2	18	0.02	4.22	15	56.69	1314.23	52,136.28	NA	NA
3	F	55	23.4	0.8	50	4.96	5.67	12.5	25.19	1045.52	39,786.74	3 (Hydroxychloroquine, MTX, Adalimumab)	1.46
4	F	25	52.3	76	106	2.57	4.35	7.8	14.27	1541.37	27,554.49	3 (Hydroxychloroquine, MTX, Sulfasalazine)	1.13
5	F	57	<20	1.4	46	3.4	4.16	16	12.51	522.76	8659.09	3 (Hydroxychloroquine, Sulfasalazine, Prednisolone)	2.36
6	F	66	413	338	53	1.81	4.92	11.2	7.75	216.77	6251.56	2 (Hydroxychloroquine, Sulfasalazine)	2.08
7	F	34	79.8	121	8	0.03	3.45	8	5.68	114.56	4420.519	2 (Hydroxychloroquine, MTX)	1.74
8	F	43	20	3.5	28	0.68	3.46	7.1	6.52	195.36	4299.64	NA	NA
9	F	70	<20	0.7	50	2.52	4.74	12.8	4.13	101.83	2033.853	0	2.67
10	F	50	137	10	25	0.25	3.78	8	4.77	163.14	6038.51	2 (Hydroxychloroquine, MTX)	2.20

RNA expressions are shown as folds compared to the median of healthy controls. RF: rheumatoid factor; Anti-CCP: anti-cyclic citrullinated peptide antibody; ESR: erythrocyte sedimentation rate; CRP: C-reactive protein; DAS28: disease activity score 28; SDAI: simple disease activity index; NSAID: nonsteroidal anti-inflammatory drug; MTX: methotrexate; NA: not available.

## Supplementary Materials

Supplementary materials can be found at http://www.mdpi.com/1422-0067/19/9/2806/s1.

## Abbreviations

lncRNA	Long noncoding RNA
PBMC	Peripheral blood mononuclear cells
RA	Peripheral blood mononuclear cells
NTT	Noncoding transcript in T cells
