# Supplementary material for: lncRNA NTT/PBOV1 Axis Promotes Monocyte Differentiation and Is Elevated in Rheumatoid Arthritis
Source: Int J Mol Sci. 2018 Sep 18;19(9):2806. doi: 10.3390/ijms19092806 (PMC6163842; doi:10.3390/ijms19092806)
Supplement: Supplementary file 1 [file ijms-19-02806-s001.zip › ijms-338015 supplementary for proof.docx]

**Table S1.** RA patient Hb and serum cytokine levels at initial diagnosis.

| **Patient No.** | **Hb (g/dL)** | **IL-1β (pg/mL)** | **TNFα (pg/mL)** | **IL-6 (pg/mL)** |
| --- | --- | --- | --- | --- |
| 1 | 9.8 | 0.08 | 6.15 | 1.37 |
| 2 | 13.6 | 49.66 | 69.11 | 67.09 |
| 3 | 10 | 25.64 | 12.45 | 10.92 |
| 4 | 11.9 | 7.53 | 20.08 | 42.85 |
| 5 | 11.2 | <0.64 | 9.70 | 8.54 |
| 6 | 13.5 | <0.64 | 0.43 | 1.44 |
| 7 | 9.7 | 59.24 | 9.15 | 18.15 |
| 8 | 11.7 | 2.47 | 6.35 | 0.78 |
| 9 | 12.5 | 0.50 | 26.38 | 1.03 |
| 10 | 13.3 | 37.48 | 25.29 | 11.99 |
